# Assessing Botulinum Toxin Effectiveness and Quality of Life in Axillary Hyperhidrosis: A One-Year Prospective Study

**DOI:** 10.3390/diseases12010015

**Published:** 2024-01-03

**Authors:** Luca Castiglione, Marius Murariu, Estera Boeriu, Ileana Enatescu

**Affiliations:** 1 Doctoral School, “Victor Babes” University of Medicine and Pharmacy Timisoara, Eftimie Murgu Square 2, 300041 Timisoara, Romania; castiglione.luca@umft.ro; 2 Department of General Surgery, “Victor Babes” University of Medicine and Pharmacy Timisoara, Eftimie Murgu Square 2, 300041 Timisoara, Romania; murariu.marius@umft.ro; 3 Department of Pediatrics, “Victor Babes” University of Medicine and Pharmacy Timisoara, Eftimie Murgu Square 2, 300041 Timisoara, Romania; 4 Department of Obstetrics and Gynecology, Discipline of Childcare and Neonatology, “Victor Babes” University of Medicine and Pharmacy Timisoara, Eftimie Murgu Square 2, 300041 Timisoara, Romania; enatescu.ileana@umft.ro

**Keywords:** hyperhidrosis, botox, quality of life

## Abstract

This study hypothesized that botulinum toxin (Botox) therapy would sustainably reduce sweat production in axillary hyperhidrosis patients over one year and significantly improve various quality-of-life aspects, including psychological well-being, social interactions, and daily functioning. The objectives were to quantitatively measure changes in sweat production and qualitatively assess the evolving impact on patients’ quality of life over one year. Conducted prospectively at the Pius Brinzeu Clinical Emergency Hospital in Timisoara, Romania, this study complied with ethical standards and included adults with primary axillary hyperhidrosis unresponsive to conventional treatments. Participants underwent Botox injections and were evaluated at baseline, six months, and one year, using the Hyperhidrosis Disease Severity Scale (HDSS), WHOQOL-BREF, and the Dermatology Life Quality Index (DLQI), among other tools. Involving 81 patients, the study showed significant improvements in sweat production and quality-of-life metrics. Sweat production decreased from 0.81 g to 0.23 g per 15 min over one year (*p* < 0.001). HDSS scores reduced from 3.4 to 1.5, indicating a decrease in symptom severity (*p* < 0.001). The DLQI total score, assessing life quality impact, notably dropped from 19.9 to 6.9 (*p* < 0.001). Quality-of-life domains also showed significant improvements, especially in the social (from 65.3 to 73.4, *p* < 0.001) and environmental aspects (from 68.0 to 72.1, *p* < 0.001). Higher HDSS and sweat production were significantly associated with a lower quality of life on the DLQI (B coefficients of −4.1 and −2.5, respectively). Botulinum toxin therapy proved effective in reducing sweat production and improving the quality of life in axillary hyperhidrosis patients over a one-year period. These improvements were statistically significant in both physical and psychosocial domains. The study highlights the potential long-term benefits of Botox therapy for hyperhidrosis.

## 1. Introduction

Axillary hyperhidrosis, characterized by excessive and often uncontrollable sweating in the axillary region, can cause significant discomfort and unpleasant situations in modern society [1,2]. This condition, while not life-threatening, poses substantial psychosocial and emotional burdens, leading to social embarrassment, anxiety, and occupational limitations [3,4]. The pathophysiology of axillary hyperhidrosis involves hyperactive eccrine sweat glands, which are primarily influenced by cholinergic sympathetic nerves [5,6]. The excessive sweating often does not correlate with thermoregulatory requirements, indicating a dysregulation within the autonomic nervous system [7].

Current therapeutic strategies for axillary hyperhidrosis range from topical agents and iontophoresis to surgical interventions [8]. However, these treatments vary in their efficacy, invasiveness, and side-effect profiles [9]. Botulinum toxin, a neurotoxin produced by Clostridium botulinum, has emerged as a promising non-invasive treatment option for hyperhidrosis [10], by inhibiting the acetylcholine release at the neuromuscular junction, thereby reducing sweat production [11]. Previous studies have demonstrated the effectiveness of botulinum toxin in managing symptoms of hyperhidrosis, with notable improvements in both sweat production and patient-reported quality of life [12].

Additionally, while the immediate physiological effects of botulinum toxin on sweat production are well-documented, its long-term psychosocial impacts are less understood [13]. Quality of life in axillary hyperhidrosis is multifaceted, encompassing emotional, social, and functional domains [14]. Understanding how botulinum toxin therapy influences these aspects over an extended period is crucial for holistic patient care and informed decision making regarding treatment options.

This study hypothesizes that botulinum toxin therapy will demonstrate sustained effectiveness in reducing sweat production in patients with axillary hyperhidrosis over a one-year period. Additionally, it is anticipated that this treatment will lead to significant improvements in various quality of life domains, including psychological well-being, social interactions, and daily functioning. The objectives of this study are to quantitatively measure the changes in sweat production post-treatment at multiple intervals and to qualitatively assess the evolving impact of these changes on patients’ quality of life over one year.

## 2. Materials and Methods

### 2.1. Research Design and Ethical Considerations

This prospective study was performed to evaluate the effects of botulinum toxin (Botox) therapy on the quality of life in patients suffering from axillary hyperhidrosis over the course of one year. The study was conducted in private healthcare settings as well as at the Dermatology Department of the Pius Brinzeu Clinical Emergency Hospital in Timisoara, Romania, in collaboration with the Victor Babes University of Medicine and Pharmacy in Timisoara. The study adhered to rigorous ethical norms, receiving approval from the Local Ethics Committee for Scientific Research. This approval was in accordance with the EU Good Clinical Practice Directive 2005/28/EC, ICH guidelines, and the principles outlined in the Declaration of Helsinki (approval number 29/06-398).

### 2.2. Inclusion and Exclusion Criteria

The inclusion criteria in the current study comprised (1)—Adult patients aged 18 years and older; (2)—A diagnosis of primary or axillary hyperhidrosis; (3)—History of axillary hyperhidrosis for at least six months prior to enrollment; (4)—Inadequate response to conventional treatments for hyperhidrosis; and (5)—Consent to enroll in the current study and provide access to medical and personal records.

The exclusion criteria were the following: (1)—Recent history of surgical interventions in the axillary region; (2)—Presence of coexisting dermatological conditions in the axillary area; (3)—Secondary hyperhidrosis; (4)—Immunocompromised status; (5)—Pregnancy or lactation; (6)—Known hypersensitivity to botulinum toxin; and (7)—Treatment for axillary hyperhidrosis received within six months before the start of the study.

Patients with secondary hyperhidrosis, that occurs as a result of another medical condition or as a side effect of medication, were excluded due to significant differences in etiology and aspects of disease compared with primary hyperhidrosis [15]. Unlike primary hyperhidrosis, which is often idiopathic and localized to specific areas like the underarms, palms, or feet, secondary hyperhidrosis generally tends to be generalized, affecting larger areas of the body and is often accompanied by other symptoms of the underlying health issue [16].

### 2.3. Variables and Procedures

The patient recruitment process started on the 1 October 2021, until 1 October 2022. The patient assessment extended across a 12-month period, with evaluations carried out initially, at 6 months, and finally one year after the treatment. The primary factors examined included demographic details like age and gender, the duration of hyperhidrosis, sweat production levels, responses from surveys, and the results of the treatment. Experienced dermatologists administered the Botox injections, adhering to up-to-date standards in dosage and technique. For each treatment session, patients received 50 U of IncoBTX-A (Xeomin^®^, Merz Pharma, GmbH & Co KGaA, Switzerland), diluted in a 5 mL solution of sterile 0.9% saline. The injections were systematically administered in the axillary region, specifically targeting the areas with the most active sweat glands, as identified by a starch-iodine test. Typically, this involves multiple injections evenly distributed in a grid pattern across the underarm area to ensure comprehensive coverage and optimal efficacy. Two sessions were scheduled: one at the onset of the study and another at the 6-month mark. Post-injection, we closely monitored for immediate side effects and provided patients with detailed aftercare guidelines.

The intensity of hyperhidrosis was evaluated using the Hyperhidrosis Disease Severity Scale (HDSS) [17]. This involved participants rating their underarm sweating on a scale: 1 indicating ‘never noticeable’, 2 as ‘tolerable’, 3 as ‘barely tolerable’, and 4 as ‘intolerable’. A score of 3 or 4 was indicative of severe hyperhidrosis, whereas scores of 1 or 2 pointed to mild or moderate conditions. A successful treatment outcome was defined as a reduction in the score from 4 or 3 to 2 or 1, signifying a notable decrease in sweating. A recurrence of symptoms was noted if there was an increase in the score by one point following treatment. Sweat production was measured quantitatively using standard filter paper, which was weighed before and after a 15 min application to the underarms. This method calculated the rate of sweat production in grams per 15 min. All measurements were conducted under controlled conditions, maintaining room temperatures at 20–22 °C and humidity levels between 55 and 60%.

### 2.4. Surveys Employed

In order to gain a thorough understanding of the participants’ experiences, we employed several established survey tools. The WHOQOL-BREF, a 26-item questionnaire, was used to assess the overall quality of life. Additionally, the Dermatology Life Quality Index (DLQI) was integrated into this study to specifically evaluate the impact of axillary hyperhidrosis and relate it to broader quality of life measures. Furthermore, a custom survey was developed to delve deeper into the day-to-day experiences of the patients with axillary hyperhidrosis. The following questions, answered on a scale from 1 to 10, were included:

1. Underarm sweating severity: How would you rate the severity of your underarm sweating? (1—no sweating, 10—severe sweating).
2. Management of sweating: How effectively can you manage the sweating associated with your axillary hyperhidrosis? (1—not effectively at all, 10—extremely effectively).
3. Impact on daily activities: To what extent does axillary hyperhidrosis affect your daily activities? (1—no impact, 10—a significant impact).
4. Mood and emotional well-being: How would you rate your overall mood and emotional well-being? (1—very poor, 10—excellent).
5. Social engagement: How comfortable are you in social activities while dealing with underarm sweating? (1—very uncomfortable, 10—very comfortable).
6. Personal relationships: How have your personal relationships (with family, friends, etc.) been impacted by your excessive underarm sweating? (1—not affected at all, 10—significantly affected).
7. Professional and educational activities: How has your condition affected your ability to work or engage in educational activities? (1—no impact, 10—a significant impact)
8. Self-confidence: How would you rate your level of self-confidence in social and professional settings? (1—very low, 10—very high).
9. Sleep quality: How has your sleep quality been affected by axillary hyperhidrosis? (1—very poor, 10—excellent).
10. Overall quality of life: How would you rate your overall quality of life in relation to axillary hyperhidrosis? (1—very poor, 10—excellent).

Participants completed these surveys at baseline, then again at 1-month post-intervention, 6 months, and finally at 12 months (following the second Botox injection). This structured approach allowed for consistent tracking and assessment over the course of the study.

### 2.5. Statistical Analysis

Data management and analysis were conducted utilizing the statistical software SPSS version 26.0 (SPSS Inc., Chicago, IL, USA). The sample size was calculated based on a convenience sampling method, with a minimum of 74 respondents at a 99% confidence level and 3% margin of error. Continuous variables were represented as mean ± standard deviation (SD), while categorical variables were expressed in terms of frequencies and percentages. To analyze the changes between more than two means of continuous variables, the ANOVA test was utilized. The chi-square test was utilized for the categorical variables. Spearman’s correlation was used to determine associations between the study variables and quality of life factors, while a linear regression model was calculated to observe the most significant factors impacting the DLQI. A *p*-value threshold of less than 0.05 was set for statistical significance. All results were double-checked to ensure accuracy and reliability.

## 3. Results

The study included 81 patients, with an average age of 29.3 years. The age distribution was fairly even across different groups, and showed the majority of patients (39.5%) being over 40 years old. Regarding gender distribution, the study had a higher proportion of women (58%) compared to men (42%). The majority of the participants resided in urban areas (59.3%), while 40.7% came from rural settings. In terms of relationship status, a majority of the patients were either in a relationship or married (64.2%), while employment status showed that most participants were working (67.9%), followed by those who were studying (14.8%).

The duration of symptoms, as measured in months, averaged 12.6, providing a timeframe for understanding the chronicity of the participants’ experiences. Finally, the severity of hyperhidrosis, assessed using the HDSS questionnaire, showed a slight skew towards more severe cases. Less severe cases (HDSS < 3) comprised 46.9% of the participants, while more severe cases (HDSS ≥ 3) accounted for 53.1%, as described in Table 1.

Initially, the severity of underarm sweating was reported at a mean score of 7.2, which significantly reduced to 5.8 by the end of the study, indicating a notable improvement (*p* < 0.001). Similarly, the management of sweating saw a significant enhancement, with the score improving from 5.3 at the start to 7.9 after a year (*p* < 0.001). The impact of the condition on daily activities also showed a marked decrease, dropping from an initial score of 7.9 to 5.8 after one year, suggesting a substantial easing of daily challenges (*p* < 0.001). However, mood and emotional well-being remained relatively stable, with a non-significant increase.

Social engagement scores improved significantly from 5.1 initially to 6.7 at one year (*p* < 0.001), indicating enhanced social interactions over time. In contrast, personal relationships appeared to deteriorate, with scores decreasing from 6.8 to 5.0 (*p* < 0.001). Professional and educational activities saw a decline in scores from 6.0 to 5.1 (*p* < 0.001), while sleep quality varied over the year but ended with a modest improvement, with the score changing from a low of 5.3 to a high of 6.4 at 6 months (*p* = 0.043). Finally, a significant enhancement was observed in the overall quality of life, with scores increasing from 5.2 to 7.0 (*p* < 0.001), as presented in Table 2.

In the physical domain, the participants initially reported an average score of 65.5, indicating a moderate level of physical well-being, which decreased to 64.3 after treatment, rising to 67.1 at 6 months, but then falling to 63.4 at 12 months (*p*-value < 0.001), suggesting a modest yet notable variation in the physical quality of life throughout the treatment and follow-up period. In the mental domain, there was a more pronounced improvement, starting from a baseline of 63.8, up to 66.4 at 6 months, and slightly decreased to 64.7 at 12 months (*p*-value 0.020) indicating a substantial and sustained positive impact on the mental well-being of the patients.

Similarly, the social domain showed remarkable improvements starting from an initial score of 65.3 to 71.6 at 6 months, and peaked at 73.4 at 12 months (*p*-value < 0.001), demonstrating that botulinum toxin therapy had a considerable and lasting enhancement on the social aspects of the patients’ quality of life. Regarding the environmental domain, the scores started at 68.0 and showed a steady rise to 72.1 at 12 months (*p*-value < 0.001), as presented in Table 3.

The DLQI total score, which assesses the impact of hyperhidrosis on life quality, showed a marked decrease dropping from 19.9 before treatment to 7.4 immediately after treatment and 6.9 at 12 months (*p* < 0.001), indicating a substantial and lasting enhancement in life quality as perceived by the patients following the treatment.

Regarding the HDSS questionnaire, which assessed the severity of hyperhidrosis symptoms, there was a significant reduction from a mean score decrease from 3.4 before treatment to 1.8 post-treatment, and 1.5 at 12 months (*p* < 0.001). Sweat production, quantified in grams per 15 min, also exhibited a significant reduction, decreasing from 0.81 g before treatment to 0.29 g after treatment. There were slight fluctuations over the follow-up period, with a minor increase at 6 months (0.38 g) and then reducing again at 12 months (0.23 g). Despite these variations, the sweat production levels at all follow-up points remained significantly lower than the baseline (*p*-value < 0.001), as presented in Table 4.

A notable negative correlation was observed between the DLQI total score and the HDSS (rho = −0.416, *p* < 0.05), indicating that a decrease in the severity of hyperhidrosis, as measured by HDSS, is associated with an improvement in the quality of life, as reflected in the DLQI scores. Similarly, there was a significant positive correlation between HDSS and sweat production (rho = 0.322, *p* < 0.05), suggesting that higher severity ratings of hyperhidrosis are associated with increased sweat production.

**Figure 1:**
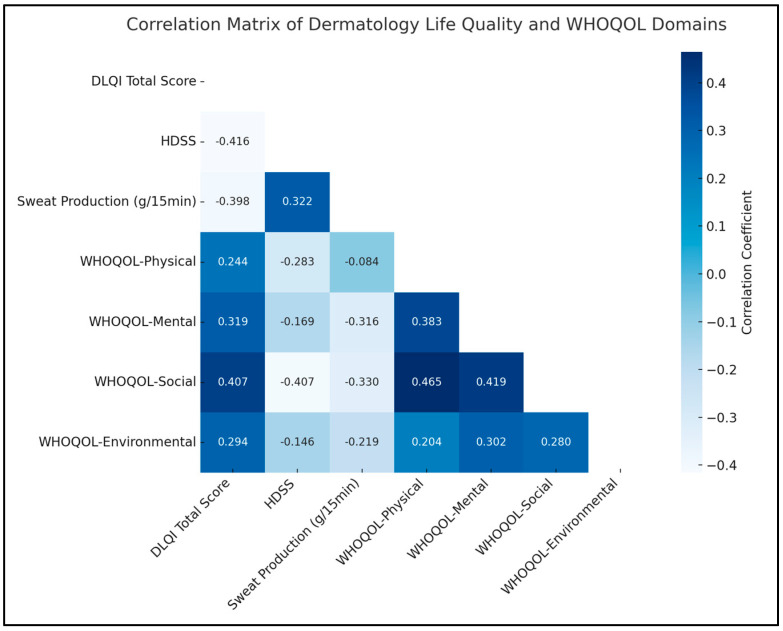
Correlation analysis.

In terms of the WHOQOL domains, a significant relationship was found between the mental domain and sweat production (rho = −0.316, *p* < 0.05), implying that lower sweat production, a key concern in hyperhidrosis, is linked to better mental quality of life. Additionally, the WHOQOL-Social domain displayed significant negative correlations with both HDSS (rho = −0.407, *p* < 0.05) and sweat production (rho = −0.330, *p* < 0.05). Therefore, lower hyperhidrosis severity and reduced sweating are associated with an enhanced social quality of life. The environmental domain also showed a significant positive correlation with the mental domain (rho = 0.302, *p* < 0.05), as presented in Table 5 and Figure 1.

The HDSS was shown to have a significant negative impact on the quality of life, evidenced by a B coefficient of −4.1 (*p* < 0.001), indicating that higher severity of hyperhidrosis is strongly associated with a lower quality of life. Similarly, sweat production, also negatively influenced the DLQI score. The B coefficient for sweat production was −2.5, with a *p*-value lower than 0.001, suggesting that increased sweat production is closely linked to a poorer quality of life.

**Figure 2:**
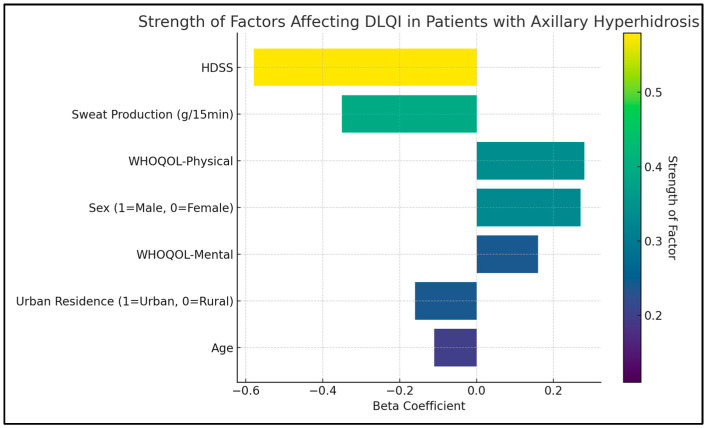
Regression analysis for DLQI total score.

Age was identified as another significant factor affecting the quality of life (B coefficient = −0.2, *p* = 0.007), suggesting that younger patients tend to report a better quality of life. The physical and mental domains of the WHOQOL survey were also significant determinants of the DLQI score. The physical domain showed a B coefficient of 0.8 (*p* = 0.021), and the mental domain had a B coefficient of 0.3 (*p* = 0.038), as presented in Table 6 and Figure 2.

## 4. Discussion

The critical findings of this study significantly enhance our understanding of hyperhidrosis treatment’s impact on patients’ lives. One of the most profound impacts observed was the reduction in underarm sweating severity, which notably decreased from a mean score of 7.2 to 5.8 over the study period. This improvement in sweating severity is a clear indicator of the efficacy of botulinum toxin in managing the physical symptoms of hyperhidrosis. Moreover, the study also highlighted significant enhancements in sweat management, as the score improved from 5.3 to 7.9 over the year. This improvement suggests that patients not only experienced less sweating but also felt more capable of managing their condition. The substantial decrease in the impact of hyperhidrosis on daily activities, from a score of 7.9 to 5.8, further underscores the treatment’s effectiveness in improving patients’ day-to-day functioning.

The study’s results indicated a differential impact on quality of life (QOL) improvements between less severe (HDSS < 3) and more severe (HDSS ≥ 3) groups with axillary hyperhidrosis. The more severe group, which initially reported higher underarm sweating severity, experienced a more pronounced decrease in their scores post-treatment, indicating significant relief from severe symptoms. Their management of sweating improved substantially, reflecting an enhanced ability to control and cope with the condition. Notably, this group’s overall quality of life, as measured by the DLQI total score, saw a marked reduction, suggesting a substantial easing of the burden of hyperhidrosis on their daily lives. Conversely, the less severe group also reported improvements, but the magnitude was comparatively lower. This could be attributed to their initially milder symptoms, which may not have allowed for as dramatic a change as seen in the more severe group. Nevertheless, both groups showed significant enhancements in their social engagement and overall quality of life scores, indicating that regardless of severity, all patients experienced a meaningful improvement in their ability to engage with others and in their general well-being.

However, the study presented a more nuanced picture of the treatment’s impact on psychological and social dimensions. While there was a modest improvement in mood and emotional well-being, this change was not statistically significant. In contrast, social engagement scores showed a significant improvement, suggesting that effective management of hyperhidrosis can lead to better social interactions and relationships. Particularly, the study highlighted the negative impact of hyperhidrosis severity measured by the HDSS and sweat production on the quality of life on the DLQI scores. Thus, reducing the severity of hyperhidrosis and sweat production is crucial for improving patients’ quality of life. Additionally, age emerged as a significant factor, indicating that younger patients might experience a more substantial impact on their quality of life due to hyperhidrosis.

Finally, the WHOQOL (physical and mental domains) were also significant determinants of the DLQI score. The positive coefficients for these domains suggest that improvements in physical health and mental well-being are associated with better overall dermatological quality of life. This finding emphasizes the need for a holistic approach in treating hyperhidrosis, addressing both the physical symptoms and the broader psychological and social effects of the condition. Moreover, even though the current study addressed only the cases of primary hyperhidrosis, patients affected by secondary hyperhidrosis from various causes [17,18,19] might have a worsened quality of life that deserves medical attention.

Numerous studies have historically documented the effectiveness of botulinum toxin type A in treating axillary hyperhidrosis [20]. A notable example was the multicentric randomized blinded study led by Heckman et al., which compared botulinum toxin type A with a placebo. Their findings revealed a significant reduction in sweat rate, from an average of 192 mg/min to 24 mg/min in the botulinum toxin type A group, in contrast to only a modest decrease to 144 mg/min in the placebo group. These results closely align with the efficacy observed in the toxin group of the present study [21]. The duration before symptom recurrence in these studies varied, ranging from 4 to 17 months. The underlying mechanisms causing recurrent hyperhidrosis post-intradermal botulinum toxin type A injection remain unclear. It is known that new nerve endings regenerate within three months following an intramuscular botulinum toxin type A injection [22]; however, the regeneration of sympathetic nerve endings, which innervate sweat glands, has not been extensively researched. Resistance to botulinum toxin type A, reported in up to 5% of patients with dystonia [23], is often attributed to the induction of antibodies against botulinum toxin type A, yet such resistance has not been observed in axillary hyperhidrosis studies. Other trials also demonstrated similar efficacy in treating axillary hyperhidrosis with varying doses of the toxin [24], and, mirroring the results of this study, no adverse events were reported.

In recent years, tumescent suction curettage has gained prominence as a surgical alternative for treating axillary hyperhidrosis [25]. This procedure, performed under local anesthesia, involves the use of a tumescent solution containing saline, bicarbonate, epinephrine, and lidocaine. The solution effectively compresses the blood vessels within the fibrous septae, significantly reducing bleeding risk. This method also boasts a lower infection rate, attributable to the open drainage technique, and the risk of hematoma formation is minimized. This minimization is due not only to mechanical compression but also to the prolonged vasoconstrictive effects of epinephrine [26].

Comparative studies examining the efficacies of surgical interventions and botulinum toxin injections in treating axillary hyperhidrosis are limited. Suction curettage, despite being a recognized surgical procedure, lacks extensive research through randomized controlled trials, especially in comparison to botulinum toxin injections. A comparative analysis between suction curettage and neurotoxin injections revealed that, three months post-treatment, neurotoxin injections were slightly more effective than suction-curettage overall, and notably more so in patients with severe hyperhidrosis [27]. Botulinum toxin, requiring repetitive intradermal injections, poses a significant financial burden, particularly as it is not subsidized by the public health insurance system in India. In this study, a direct comparison was made between subcutaneous curettage and botulinum toxin injections, administering different treatments to each axilla in the same patient. Other studies demonstrated that both modalities significantly reduced sweat rates with comparable efficacy, therefore decreasing the levels of social stigma due to excessive sweating [28]. This was in line with another study [29], which reported a 75% reduction in sweat rates following suction curettage, similar to the effects of botulinum results. Additionally, another study [30] compared different suction curettage and concluded that sharp suction curettage yielded the highest reduction in sweat rates. Our study, utilizing a three-hole liposuction cannula, achieved similar efficacy without any adverse events.

In terms of treatment-related adverse events, both botulinum toxin injections were associated with minimal complications, according to the existing literature [31]. Notably, the minor adverse events were primarily linked to subcutaneous curettage. Beyond the expected physiological scarring, patients can experience post-operative ecchymoses, and one of them developed bridle formation [32]. Similarly, patient satisfaction was high, with no lasting complaints regarding either procedure, indicating the acceptability and safety of both treatments.

Similarly to our study, one meta-analysis including participants retrospectively analyzed the effectiveness of botulinum toxin injections in treating focal hyperhidrosis, comparing them with placebo in patients with primary or secondary focal hyperhidrosis. The study’s outcomes focused on the reduction in sweat rate, severity of the disease, and life quality improvements. Results showed that Botox injections were significantly more effective than placebo in reducing sweat production with over 50%, lowering disease severity with more than 2 points on the HDSS scale (similarly to our findings), and improving the DLQI with a mean change of −5.55 [12]. Despite these positive outcomes, the evidence was considered moderate in quality and limited to short-term effects, only assessing up to eight weeks, while our study expanded this follow-up time up to 12 months.

In a comparative assessment of treatment outcomes, the botulinum toxin arm demonstrated a statistically significant superiority in achieving excellent quality of life improvements, particularly for severe cases (HDSS 4), with a 96% success rate compared to only 4% in the iontophoresis group [33]. This stark contrast was further evidenced in the cross-over cases, where 75% of patients initially treated with iontophoresis showed excellent improvement when switched to botulinum toxin, as opposed to only 16% experiencing similar benefits when the treatment direction was reversed. Notably, the duration and sustainability of improvements also varied, with botulinum toxin showing a rapid onset and a maintained effect up to 4 months, suggesting a more impactful and lasting enhancement of patient quality of life. These data underscore the potential of botulinum toxin as a more efficacious treatment modality for severe hyperhidrosis, reflecting its significant role in enhancing patient outcomes.

Therefore, while botulinum toxin type A offers a non-invasive, effective solution with a good safety profile, alternatives like tumescent suction curettage present a viable option for patients allergic to Botox or for those seeking a more permanent solution. The choice of treatment ultimately depends on individual patient preferences, severity of symptoms, and the presence of any contraindications to either method. The evidence from both pharmacological and surgical interventions underscores the importance of a tailored approach to managing this condition.

This study’s limitations include its relatively small sample size and the single-center design, potentially limiting the generalizability of the results to broader populations with axillary hyperhidrosis. The exclusion of patients with secondary hyperhidrosis or recent surgical interventions in the axillary region may have further narrowed the study’s applicability. Additionally, the reliance on self-reported survey data for quality-of-life assessments introduces a degree of subjectivity, which might not fully capture the nuances of each patient’s experience. The controlled setting for sweat production measurements, although rigorous, might not accurately reflect real-world conditions where environmental factors vary. Furthermore, the study’s one-year duration, while providing useful long-term data, does not account for the potential long-term efficacy and side effects beyond this period.

## 5. Conclusions

This study successfully validated the hypothesis that botulinum toxin (Botox) therapy offers a sustainable reduction in sweat production and significantly improves the quality of life for patients with axillary hyperhidrosis over a one-year period. The comprehensive analysis of 81 patients demonstrated notable improvements across all measured parameters. Sweat production significantly decreased, symptom severity reduced, and the overall impact on quality of life markedly lessened. Notably, the social and environmental domains of patients’ lives saw substantial enhancements, reflecting the broader positive implications of this therapy. The significant association between higher HDSS and sweat production with a lower quality of life underscores the effectiveness of Botox in addressing both the physical and psychosocial challenges of hyperhidrosis. Overall, the study firmly establishes the long-term benefits of Botox therapy in improving daily functioning and well-being in individuals suffering from this condition, providing a robust evidence base for its continued use and further exploration in clinical practice.

## Figures and Tables

**Table 1:** Patients’ personal history.

Variables	*n* = 81	%
Age (mean ± SD)	29.3 ± 8.1	-
18–30 years	26	32.1%
30–40 years	23	28.4%
>40 years	32	39.5%
Sex		
Men	34	42.0%
Women	47	58.0%
Area of residence		
Urban	48	59.3%
Rural	33	40.7%
Relationship status		
Single/Divorced	29	35.8%
In a relationship/Married	52	64.2%
Work/study status		
Working	55	67.9%
Studying	12	14.8%
None of the above	14	17.3%
Symptom duration, months (mean ± SD)	12.6 ± 7.7	-
Hyperhidrosis severity		
Less severe (HDSS < 3)	38	46.9%
More severe (HDSS ≥ 3)	43	53.1%

SD—Standard Deviation; HDSS—Hyperhidrosis Disease Severity Scale.

**Table 2:** Evaluation of patient-reported quality of life outcomes over a 1-year period.

Questions	Before	After	6 Months	1 Year	*p*
1. Underarm sweating severity	7.2 ± 2.8	6.4 ± 2.3	5.7 ± 2.6	5.8 ± 3.2	0.001
2. Sweating management	5.3 ± 1.1	6.3 ± 3.3	7.2 ± 2.5	7.9 ± 1.9	<0.001
3. Impact on daily activities	7.9 ± 3.0	7.1 ± 3.2	5.1 ± 3.1	5.8 ± 2.9	<0.001
4. Mood and emotional well-being	6.5 ± 2.6	6.6 ± 3.4	6.0 ± 2.5	6.9 ± 2.7	0.248
5. Social engagement	5.1 ± 2.5	5.9 ± 2.4	6.4 ± 2.1	6.7 ± 3.0	<0.001
6. Personal relationships	6.8 ± 1.1	6.7 ± 2.6	5.5 ± 2.0	5.0 ± 1.2	<0.001
7. Professional and educational activities	6.0 ± 1.2	6.2 ± 2.0	5.3 ± 1.8	5.1 ± 1.6	<0.001
8. Self-confidence	5.6 ± 2.6	5.9 ± 1.5	6.0 ± 2.2	6.3 ± 1.3	0.159
9. Sleep quality	5.8 ± 3.9	5.3 ± 2.2	6.4 ± 1.6	5.9 ± 2.1	0.043
10. Overall quality of life	5.2 ± 1.2	5.1 ± 2.8	6.8 ± 3.0	7.0 ± 2.5	<0.001

Data presented as Mean ± Standard Deviation (interpretation of results described in the Section 2).

**Table 3:** Longitudinal assessment of WHOQOL-BREF survey results.

WHOQOL-BREF	Before	After	At 6 Months	At 12 Months	*p*
Physical domain	65.5 ± 5.4	64.3 ± 5.2	67.1 ± 5.0	63.4 ± 4.8	<0.001
Mental domain	63.8 ± 4.9	65.3 ± 5.9	66.4 ± 5.6	64.7 ± 5.1	0.020
Social domain	65.3 ± 4.1	66.9 ± 4.0	71.6 ± 5.6	73.4 ± 6.0	<0.001
Environmental domain	68.0 ± 4.6	69.7 ± 4.2	70.2 ± 4.3	72.1 ± 5.0	<0.001

Data presented as Mean ± Standard Deviation; WHOQOL-BREF—Brief Version of the World Health Organization Quality of Life survey (higher scores indicate better quality of life).

**Table 4:** Longitudinal assessment of DLQI survey results in association with HDSS and sweat production.

Variables (Mean ± SD)	Before	After	At 6 Months	At 12 Months	*p*
DLQI total score	19.9 ± 5.3	7.4 ± 3.0	6.6 ± 2.8	6.9 ± 3.1	<0.001
HDSS	3.4 ± 0.8	1.8 ± 0.5	1.7 ± 0.7	1.5 ± 0.9	<0.001
Sweat production (g/15 min)	0.81 ± 0.26	0.29 ± 0.33	0.38 ± 0.10	0.23 ± 0.17	<0.001

ANOVA test; SD—Standard Deviation; DLQI—Dermatology Life Quality Index (higher scores indicate lower quality of life); HDSS—Hyperhidrosis Disease Severity Scale.

**Table 5:** Correlation matrix.

Variables (rho, *p*-Value)	DLQI Total Score	HDSS	Sweat Production (g/15 min)	WHOQOL-Physical	WHOQOL-Mental	WHOQOL-Social	WHOQOL-Environmental
DLQI Total Score	1						
HDSS	−0.416 *	1					
Sweat Production (g/15 min)	−0.398	0.322 *	1				
WHOQOL-Physical	0.244	−0.283	−0.084	1			
WHOQOL-Mental	0.319	−0.169	−0.316 *	0.383 *	1		
WHOQOL-Social	0.407	−0.407 *	−0.330 *	0.465 *	0.419 *	1	
WHOQOL-Environmental	0.294	−0.146	−0.219	0.204	0.302 *	0.280	1

*—Statistically significant associations (*p* < 0.05); Spearman’s rho is used due to the ordinal nature of some variables.

**Table 6:** Regression analysis for DLQI determinants in patients with axillary hyperhidrosis.

Variables	B Coefficient	Standard Error	Beta	*t*-Value	*p*-Value
Constant	13.8	2.2	-	9.61	<0.001
HDSS	−4.1	0.7	−0.58	−5.86	<0.001
Sweat Production (g/15 min)	−2.5	0.6	−0.35	−4.17	<0.001
Age	−0.2	0.1	−0.11	−1.22	0.007
Sex (1 = Male, 0 = Female)	1.1	0.9	0.27	1.91	0.282
Urban Residence (1 = Urban, 0 = Rural)	−0.4	0.6	−0.16	1.05	0.319
WHOQOL-Physical	0.8	0.2	0.28	1.37	0.021
WHOQOL-Mental	0.3	0.1	0.16	1.25	0.038

Dependent Variable—DLQI Total Score; B: Unstandardized regression coefficient; Beta: Standardized regression coefficient.

## Data Availability

Data available on request.
